# Factors associated with risky sexual behaviour among secondary and preparatory students in Wolaita Sodo town, Southern Ethiopia; Institution based cross-sectional study

**DOI:** 10.4314/ahs.v21i4.41

**Published:** 2021-12

**Authors:** Gedion Asnake Azeze, Natnael Atnafu Gebeyehu, Addisu Yeshambel Wassie, Taklu Marama Mokonnon

**Affiliations:** Department of Midwifery, College of Health Science and Medicine, Wolaita Sodo University, Wolaita Sodo, Ethiopia

**Keywords:** Students, risky sexual behavior, factors, Ethiopia

## Abstract

**Background:**

Young people in Ethiopia aged between 18 and 24 who have had sex before age 18 has increased from 35% in 2005 to 40% in 2016 among women and from 9% to 12% among men.

**Objectives:**

This study aimed to assess the prevalence and factors associated with risky sexual behavior among secondary and preparatory students in Wolaita Sodo town, Wolaita zone, Southern Ethiopia; 2020.

**Methods:**

A school-based cross-sectional study was conducted from February 4 to 25, 2020. Multistage sampling was employed to select a random sample of 830 study participants from 2 randomly selected secondary and preparatory schools. Bivariate and multivariate logistic regression analysis was used to examine the relationship between the outcome variables and independent variables.

**Result:**

Among the 306 participants who reported ever having sex, 196 (24.7%) engaged in risky sexual behaviors. Factors significantly associated with risky sexual behavior were; ever used alcohol, ever smoked cigarettes, parent monitoring, and having sexually active close friend/s.

**Conclusion:**

Substantial proportion of study participants engaged in risky sexual behavior calling for more interventions on school student's addictive behaviors. Parents should have frequent, open and informative discussions about substance use and the associated problems with their adolescents.

## Introduction

As widely understood, sexual health is a state of physical, mental, emotional, and social well-being in relation to sexuality^1^. The recognition of the different forms of sexual behaviors and their expressions contributes to people's overall sense of well-being and health and, thus, needs to take a central position in the heart of public health policymakers and researchers^1^,^2^. Sexual risk behaviors, as defined by World Health Organization (WHO), are sexual activities that increase the probability of adverse sexual and reproductive health including unwanted pregnancy, unsafe abortion, Sexually Transmitted Infections (STIs) including HIV/AIDS and others^3^. These sets of behaviors may also include premarital sex, having risky casual or unknown sexual partners, having concurrent multiple sexual partners, failure to discuss risk topics before intercourse, and engaging in unprotected sexual activities under the influence of substances that may lead to harmful and unintended health outcomes^4^–^7^.

Risk-taking behavior is on the rise all over the world8. In particular, sexual activities among youth and young adults have been reported to be increasing worldwide putting them at higher risk than other age-groups for varying reproductive health problems ranging from unwanted pregnancy to abortion, to contracting sexually transmitted diseases (STDs) including HIV infection and even to death^9^. There has been increasing public health concern about the reducing age of adolescent's initiation of sexual activities. Each year, approximately one million young women aged 15–19 become pregnant; the vast majority of these pregnancies are unplanned^10^,^11^. Furthermore, the international monitoring data indicate that 70% of patients with STI are aged between 15 and 24 years and the WHO estimates that one out of 20 teenagers contracts an STI each year^12^.

Previous studies conducted among adolescents in sub-Saharan Africa have documented increasing premarital sexual activities which can be associated with undesired consequences such as illegal abortion, risk of HIV infection and school dropout related to pregnancy^6^,^13^–^15^. A recent qualitative study conducted in Nigeria reported that from Focused Group Discussion (FGD), parents, health care professionals and females in school or out of school estimated seven out of ten females will get pregnant due to unsafe sex and from these; three to four females out of five will look for local methods to terminate the pregnancy^16^.

Having large networks of friends or the opposite sex of friends at schools may affect adolescents and young adult's sexual behavior either positively or negatively^17^,^18^. A growing body of literature shows adolescents and young adults in Ethiopia have been found to be at the highest risk for reproductive and sexual health problems including unwanted pregnancy, unsafe abortion and sexually transmitted infections including HIV^10^,^19^,^20^. Recently, the Ethiopian Demographic and Health Survey (EDHS) reported that the percentage of first sexual intercourse among young people in Ethiopia aged between 18 and 24 who have had sex before age 18 has increased from 35% in 2005^21^ to 40% in 2016^22^ among women and from 9% to 12% among men. Likewise, according to 2011 EDHS, 29% of women had first sexual intercourse before age 15 years old and 62% of women before the age of 18 years old^14^. Regrettably, adolescents are said not to use condoms thus, placing them at risk of unplanned pregnancies, STIs/HIV and related adverse health consequences^12^,^23^.

Given the fact that Ethiopia, a country where sexual matters are very sensitive and less likely to be discussed openly, this study is therefore tasked to assess the magnitude and factors associated with risky sexual behavior among secondary and preparatory students in Sodo town, Wolaita zone, Southern Ethiopia. More importantly, findings from this study are expected help planners, administrators, and stakeholders in the field of sexual and reproductive health to modify strategy, thereby, on one hand, improving youth SRH clinic service utilization, and decreasing the probability of adverse sexual and reproductive health problems on the other. It will also generate additional research questions for future study.

## Methods and materials

### Study setting and design

Institution based cross-sectional study was conducted from February 4 to 25, 2020 among high school and preparatory going students in Wolaita Sodo, Southern Ethiopia. Sodo town is the capital town of the Wolaita zone, one of the 12 zones in Southern Nations, Nationalities and Peoples' Region (SNNPR) with an area of 830 square kilometers. The study was conducted among regular day time 9th -12th grade students. The population structure consists of mainly youth. Evidence from Wolaita Sodo town education office shows the total number of secondary and preparatory students in Wolaita Sodo town is 2181 Females and 4685 Males. The town has 4 secondary and preparatory schools, 1 Technical and Vocational Training College (TVET), and 1 University (Wolaita Sodo University).

### Source and Study population

All regular secondary and preparatory school students in Wolaita Sodo town enrolled during the 2019/20 academic year were considered as a source of the population while those students attending their class during the study period in selected secondary and preparatory schools were considered as study populations for this study. Students who were learning in the Nightshift and those who will not be able to complete the questionnaire without assistance (having visual, hearing impairments and seriously ill) were excluded from the study.

### Sample size determination and sampling procedure

The sample size was determined using the prevalence of risky sexual behavior and important factors associated with risky sexual behavior. Factors associated with risky sexual behavior from previously conducted studies^17^,^24^,^26^ which were considered to determine the sample size for the current study include: watching a pornographic movie, substance use, peer influence, not using reproductive health clinics, and had no parental control. The maximum sample size obtained was by using single population proportion formula based on assumptions including; proportion of risky sexual behavior 43.1%^24^, 95% confidence level, 5% margin of error, and 10% for an anticipated non-response rate of the respondents. Hence, after taking a design effect of to, a total of 830 participants were considered for this study.

Multistage sampling technique was used to select study participants from each selected schools. After identifying and listing all secondary and preparatory schools (9th to 12^th^ grade) in Wolaita Sodo town, two schools (one from private and one from governmental) were selected by lottery method from a total of four secondary and preparatory schools (three private and one governmental). The total calculated sample size was proportionally allocated to selected schools, and sample proportional to size allocated for each grade. Finally, sections that could accommodate the allocated sample to each school were selected by using a lottery method, and simple random sampling technique was used for selecting participants from each grade after preparing the sampling frame using a list of the names of the students from the class attendances. Randomly selected students were notified through the student representative and they were then provided orientation in their classrooms about the study, how they were selected, and confidentiality issues. Students that were willing to participate were provided with the questionnaire. A similar method in selecting institutions and recruiting study participants has been applied in previously conducted studies^10^,^17^,^26^.

### Measurements

Data was collected by using a pre-tested, structured, self-administered questionnaire that was adopted and modified according to local context and the objectives of the study from previous literature on a similar topic. The questionnaire was initially developed in English and translated to Amharic (local language) and then back to English by different language experts to check for meaning consistency. Respondents were asked about socio-demographic characteristics, substance use and related characteristics, parent-related characteristics, STIs related characteristics, and sexual behaviors and related characteristics. Substance use (cigarette smoking, alcohol consumption and chew khat) were measured using a question ‘Have you ever smoked cigarette/drunk alcohol/chew khat?’. Inconsistent use of condoms, having multiple sexual partners and having sexual intercourse with (or for female participants, being act as) commercial sex workers were measured over the 12 months prior to data collection.

### Study Variables

Dependent variable: Risky sexual behavior.

Independent Variable: (i) Socio-demographic variables; Age, sex, marital status, religion, ethnicity, grade level, history of school drop, living arrangement, and support source. (ii) Parent related characteristics: Father's highest education level, mother's highest education level, father's occupation, mother's occupation, friends are known by parents, perception of family's economic status, and parental monitoring. (iii) Sexual behaviors and related characteristics: Ever had sexual intercourse, Age at first sexual intercourse, reasons for having sexual intercourse, length of time respondent knew their sexual partner, having sexual intercourse during the previous 12 months, and having sexually ative close friend/s.

### Data Processing and Analysis

During data collection period, questionnaire completeness was checked daily by the supervisors. Data were coded and entered into EpiData Entry software (version 3.1, EpiData Association, Odense, Denmark) and analyzed by using STATA window version 15.0 (StataCorp. LP, College Station, TX, USA). Descriptive statistics were done. Both bivariate and multivariable logistic regression models were used to identify associated factors. Any variable which in bivariate analysis had a P-value <0.2 was put into a multivariate model to adjust for confounders using logistic regression. Levels of significance were set at 5% (P<0.05).

### Data quality assurance

Pre-testing of the questionnaire was conducted on 5% of the sample (among 42 students) attending one secondary and preparatory school that was not selected for the study. Corrections regarding logic and wording of the questionnaire were noted and corrected before conducting the actual data collection. Furthermore, one day training was given to orient data collectors and supervisor on the questionnaire to be used, the purpose of the study and how to approach respondents and obtain consent. Appropriate supervision for data collectors have been made.

### Operational and term definitions

The outcome variable, risky sexual behavior, were a composite outcome variable which includes reporting one or more off the following; early sexual debut, inconsistent use of condoms, having multiple sexual partners, and/or having sexual intercourse with (or for female participants, being act as) commercial sex workers. In the present study, the Cronbach's alpha value was 0.821. This procedure is consistent with established methodologies for assessing risky sexual behavior^2^. Parental monitoring has been measured by adolescents' perceived parental knowledge of where they go and what they do, along with the amount of unrestricted or unsupervised time that they experience^25^

### Ethical considerations

This study was approved by Wolaita Sodo University, College of Health Science and Medicine Ethical Review Committee (CHSM/ERC/104), and ethical clearance was obtained from the same office. Support letter was written to selected Secondary and Preparatory Schools explaining the objectives of the study and its significance so that directors of selected schools were contacted and requested permission to conduct the survey in their school. Participants were informed about the purpose, benefit, risk, confidentiality of the information and the voluntary nature of participation in the study. For students aged 18 years and above, informed own consent was taken. For students less than 18 years, informed consent was obtained from parents and in case of parents with no formal education, informed consent was obtained from legally authorized representatives of the minors and respondents' informed written assent was obtained to collect data. Participants were informed that they had the right to withdraw from the study at any time. All methods were performed following the relevant guidelines and regulations.

## Result

### Socio-demographic characteristics

Out of the total 830 study participants, 794 of participants took part in the survey with a response rate of 95.7%. The mean age of the participants was 18.2 years (SD ± 2.5 years) with a range of 13 to 30 years and majority (75.9%) of them belong to the age group of 15 to 19 years. About 329(41.4%) were females and almost three quarters 601(75.7%) were Wolaita followed by Amhara 104(13.1%) by ethnicity and 464(58.4%) were living with parents. Furthermore, majority of the students, 694 (87.4%) were single, 400 (50.4%) were protestant by religion, and more than three fourth (78.6%) had no history of school drop (Table 1).

**Table 1:** Socio-demographic characteristics of school students in Wolaita Sodo town, Wolaita zone, Southern Ethiopia; 2020

Variables	Frequency (n)	Percentage (%)
**Age**		
<15	20	2.5
15–19	603	76.0
20–30	171	21.5
**Marital status**		
Single	694	87.4
Married	75	9.5
Others (cohabited, divorced)	25	3.1
**Religion**		
Protestant	400	50.4
Orthodox Christian	276	34.8
Muslim	67	8.4
Adventist	51	6.4
**Ethnicity**		
Wolaita	601	75.7
Amhara	104	13.1
Oromo	47	5.9
Kembata	18	2.3
Others (Tigre, Hadiya, Sidama)	24	3.0
**Grade level**		
Grade 9	251	31.6
Grade 10	209	26.3
Grade 11	192	24.2
Grade 12	142	17.9
**History of school drop**		
No	624	78.6
Yes	170	21.4
**Living arrangement**		
With parents/family	464	58.4
Alone	217	27.4
With friend/s	113	14.2
**Supported by**		
Parent	573	72.2
Self	127	16.0
Voluntary organizations	66	8.3
Relatives/friends	28	3.5

### Parent related characteristics

Concerning father's highest education level, one hundred thirty (16.4%) did not attend formal education while less than one fifths (19.1%) highest educational level was college and above. Around thirty-eight percent of the participant's perception of family's economic status belong under the category poor and those who responded to medium economic status were little less than half percent (44.3%). 639 (80.5%) and 573(72.2%) of participant's fathers and mothers respectively, were alive during the data collection period. Of all, 681(85.8%) of respondents reported their parents monitor them where they are, with whom they stay and what they were doing when they leave home out of school time (Table 2).

**Table 2:** Parent related characteristics of school students in Wolaita Sodo town, Wolaita zone, Southern Ethiopia; 2020

Variable	Frequency (n)	Percent (%)
**Father's highest education level**		
No formal education	130	16.4
Primary education	271	34.1
Secondary education	241	30.4
College and above	152	19.1
**Mother's highest education level**		
No formal education	143	18.0
Primary education	252	31.0
Secondary education	238	30.0
College and above	161	20.3
**Father's occupation**		
Government employee	282	35.5
Merchant	242	30.5
Farmer	167	21.0
Private employee	77	9.7
Others^a^	26	3.3
**Mother's occupation**		
Government employee	114	14.4
Merchant	362	45.6
Housewife	166	20.9
Private employee,	104	13.1
Students	29	3.6
Handicraft	19	2.4
**Perception of family's economic status**		
Poor	300	37.8
Medium	352	44.3
Rich	142	17.9
**Parental monitoring**		
Yes	681	85.8
No	113	14.2
**Friends are known by parents**		
Yes	615	77.5
No	179	22.5

a fishers, tailor and taxi driver

### Sexual behaviors and related characteristics

**Figure 1:**
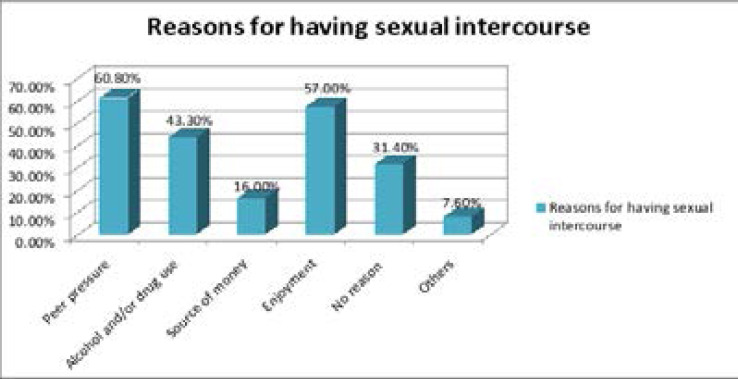
Reasons for having sexual intercourse mentioned by school students in Wolaita Sodo town, Wolaita zone, Southern Ethiopia; 2020.

Our study finding showed that 306(38.5%) of study participants had ever had sexual intercourse out of them, 104(34.0%) were females and the rest 202(66.0%) were males. Age of first sexual encounter ranges from 14 to 27 years with the mean age of 18.2(±1.7 SD) years. Peer pressure (60.8%), sex for enjoyment (57%), and alcohol and/or drug use (43.3%) were the main reasons for indulging in sex among the respondents (Figure 1).

**Figure 2:**
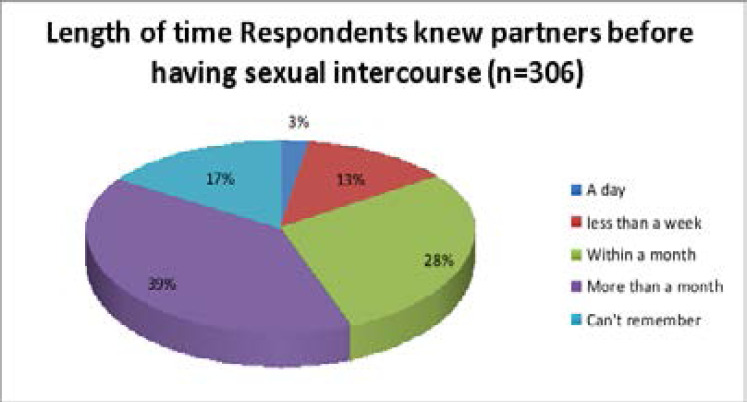
Length of time respondents knew partners before having sexual intercourse among school students in Wolaita Sodo town, Wolaita zone, Southern Ethiopia; 2020.

Thirty-nine percent of participants knew their sexual partner for more than a month while little less than one fifth (17%) of participants can't remember the length of time they knew their sexual partner before having sexual intercourse (Figure 2). Out of those who ever had sexual intercourse, two hundred and forty (30.2%) had sexual intercourse during the previous 12 months. Little more than half 432(54.4%) of study participants had sexually active close friend/s.

The outcome variable, risky sexual behavior, was generated from a composite variables as practicing either of; early sexual debut, inconsistent use of condom, having multiple sexual partners, and having sexual intercourse with (or for female participants, being act as) commercial sex workers. With regards to early sexual debut, one in five (20.9%) of the study participants who had ever sexual intercourse claimed they were sexually active at an early age (<18 years). On the other hand, out of those who had sexual intercourse in the last 12 months prior to the study, 34(14.8%), 77(32.1%), and 120(50.0%) reported having sexual intercourse with (or for female participants, being act as) commercial sex worker, having multiple sexual partners, and inconsistent use of condom respectively. As a result, the overall magnitude of risky sexual behavior in this study is 196 (24.7% with 95% CI: 21.8, 27.8).

### Substance use and related characteristics

About 313(39.4%), 132(16.6%), 490(61.7%), 114(14.4%), 54(6.8%), and of participants reported they ever; drunk alcohol, attended night club, watched pornographic film, chew khat (an evergreen plant locally produced psycho-stimulant), and smoked cigarette respectively. Furthermore, more than half (432) and around twenty-one percent (170) of participants reported having sexually active close friends and chew khat respectively. Sexually transmitted infections related characteristics: Almost all, 774(97.5%) of respondents explained that they heard the term sexually transmitted infection and from these, majority (81.0%) of them have got information from radio or television followed by friends (41.6%) and books or health magazines (19.9%) (Table 3).

**Table 3:** Sexually transmitted infections related characteristics of school students in Wolaita Sodo town, Wolaita zone, Southern Ethiopia; 2020

VARIABLES	FREQUENCY (n)	PERCENT (%)
**Heard about STIs**		
Yes	774	97.5
No	20	2.5
**Sources of information**		
Radio/television	643	81.0
Friends	330	41.6
Books/health magazines	158	19.9
Health staff	133	16.8
Anti-HIV/AIDS clubs	105	13.2
Internet	94	11.8
Parents	59	7.4
**STDs vaccine availability**		
Some have vaccine, some do not have	151	19.0
Every STD has vaccine	101	12.7
No STD has vaccine	348	43.8
Unknown	194	24.5
**STIs can be cured**		
Cannot be cured	304	38.3
Can be cured	490	61.7

### Factors associated with risky sexual behavior

Bivariate analysis was done to assess any association between independent variables and risky sexual behavior. Accordingly, variables which made an association in bivariate analysis with P-value <0.2 were; sex, age, history of school drop, participant's mothers alive, parent monitoring, having sexually active close friend/s, perception of family's economic status, ever smoked cigarette, ever used alcohol, curability of STDs, and vaccine for STDs (Table 4). After controlling for confounders through multivariable logistic regression, ever used alcohol, ever smoked cigarettes, parental monitoring, and having sexually active close friend/s were found to be significantly associated (p<0.05) with risky sexual behavior among study participants (Table 4).

**Table 4:** Bivariate and multivariate analyses of factors associated with risky sexual behavior among school students in Wolaita Sodo town, Wolaita zone, Southern Ethiopia; 2020

Variable	Risky Sexual Behavior		Crude OR (95%C.I)	Adjusted OR (95%C.I)
	No (%)	Yes (%)		
**Sex**				
Male	342(73.5)	123(26.5)	1	1
Female	256(77.8)	73(22.2)	0.79(0.56, 1.10)	0.89(0.61, 1.29)
**Age**				
<15	19(79.2)	5(20.8)	0.65(0.23, 1.85)	0.19(0.02, 1.54)
15–19	454(76.3)	141(23.7)	0.77(0.53, 1.13)	0.86(0.56,1.31)
20–30	125(71.4)	50(28.6)	1	1
**History of school drop**				
No	481(77.1)	143(22.9)	1	1
Yes	117(68.8)	53(31.2)	1.52(1.04, 2.21)	1.40(0.92, 2.13)
**Parental monitoring**				
Yes	525(77.1)	156(22.9)	1	1
No	73(64.6)	40(35.4)	1.84(1.20, 2.82)	1.86(1.16, 3.00)
**Participants' mother alive**				
Yes	439(76.6)	134(72.0)	1	1
No	159(64.6)	62(28.0)	1.27(0.89, 1.81)	1.37(0.93, 2.03)
**Having sexually active close** **friend/s**				
No	293(81.0)	69(19.0)	1	1
Yes	305(70.6)	127(29.4)	1.76(1.26, 2.46)	1.59(1.10, 2.31)
**Perception of family's** **economic status**				
Poor	231(77.0)	69(23.0)	1	1
Medium	272(77.3)	80(22.7)	0.98(0.68, 1.42)	0.97(0.65, 1.46)
Rich	95(66.9)	47(33.1)	1.65(1.06, 2.57)	1.41(0.86, 2.30)
**Ever smoked cigarette**				
No	572(77.3)	168(22.7)	1	1
Yes	26(48.1)	28(51.9)	3.66(2.09, 6.42)	2.05(1.10, 3.83)
**Ever drink alcohol**				
No	418(86.9)	63(13.1)	1	1
Yes	180(57.5)	133(42.5)	4.90(3.46, 6.93)	4.27(2.95,6.18)
**STIs can be cured**				
No	220(72.4)	84(27.6)	1	1
Yes	378(77.1)	112(22.9)	0.77(0.55, 1.07)	0.87(0.60, 1.26)
**Vaccine for STIs**				
Some have and some do not	122(80.8)	29(19.2)	1	1
Every STIs have vaccine	80(79.2)	21(20.8)	1.10(0.58, 2.07)	1.01(0.50, 1.98)
No STIs have vaccine	268(77.0)	80(23.0)	1.25(0.78, 2.02)	1.06(0.62, 1.78)
Unknown	128(66.0)	66(34)	2.16(1.31, 3.58)	1.72(0.98, 3.02)

The odds of reporting risky sexual behavior was nearly four and two times higher among participants whoever drank alcohol (AOR = 4.27; 95% CI: 2.95, 6.18) and ever smoked cigarette (AOR = 2.05; 95% CI: 1.10, 3.83) respectively. Moreover, risky sexual behavior was significantly associated with having sexually active close friend/s in which, the odds of reporting risky sexual behavior was 60% higher among participants who had sexually active close friend/s (AOR = 1.59; 95% CI: 1.10, 2.31). Participants who had no parental monitoring were nearly 90% more likely (AOR = 1.86; 95% CI: 1.16, 3.00) to be engaged in risky sexual behavior compared to their peers.

## Discussion

This study was aimed to assess the prevalence and factors associated with risky sexual behavior among school students in Wolaita Sodo town, Ethiopia. Accordingly, nearly one out of four students (24.7% with 95% CI of 21.8 – 27.8) fulfilled the operational criteria of risky sexual behavior. This finding is congruent with previous studies; Haramaya town (25.3%)^26^ and Addis Ababa, Ethiopia (26.7%)10. The observed similarity between these findings might be due to the fact that studies were conducted in the same country and it's believed that these towns may not have significant difference in their socio-demographic characteristics.

Moreover, our review of literature shows that the magnitude of RSB was higher than some other studies conducted; at Bahirdar town, Ethiopia (19%)^27^ and Sri Lanka (12.4%)28. The observed variation in finding between these studies may be explained by the difference in methodologies and socio-economic status. For instance, the study at Bahirdar town was conducted by taking only one institution and a relatively small sample of adolescents whose age is between 14 and 19 years. However, participants in our study were more aged ranging from 13 to 27. This explanation was further supported by a study which documented that prevalence of sexual experience amongst youth seems to increase with age^29^. As well, the possible reason for the difference in finding between studies conducted at Ethiopia^27^ and Sri Lanka^28^ might have to do with difference on cut point (for the study conducted at Sri Lanka, authors defined RSB as reporting of one or more out of three behaviors), the nature of questions that respondents were asked, and study population from different cultural and socio-demographic background.

On the other hand, the magnitude of RSB in our study is far less than findings from Addis Ababa, (43.1%)^24^ Jimma zone, Southwest Ethiopia (42.1%)4 and college students of Lusaka, Zambia (72.2%)^30^. The observed variation in findings might be due to methodological and study participant's differences across areas and between studies. From aforementioned studies, for instance, a comparative cross-sectional study was conducted among youth center reproductive health clinics users and non-users on study conducted at Addis Ababa^24^ and mixed-method (qualitative and quantitative approaches) on a sample of 271 students was conducted in selected preparatory schools (11^th^ and 12^th^ grade students) at Jimma zone, Southwest Ethiopia4. Further, this may also be due to difference in exposure status of the youth which could be related to the fact that youth in the capital city of Ethiopia (Addis Ababa) is believed more likely to be involved in risky sexual behavior due to urbanization, the influence of mass media, the rapid development of the economy and degradation of traditional values.

### Factors associated with risky sexual behavior

Findings from this study revealed that alcohol consumption strongly associated with risky sexual behavior in which; the odds of reporting RSB among participants who drank alcohol were little more than four times than their counterparts. Similar finding has already been observed in other studies conducted locally at West Gojjam Amhara, Ethiopia^2^ and studies conducted outside of Ethiopia; at Sri Lank^28^ and Central Tanzania31 which also reported that those who consumed alcohol were 2.6 and 2.8 times more likely to be involved in risky sexual behavior compared to non-users. This could be related to the fact that consuming alcohol and drugs would increase risk-taking behavior, impair one's judgment on the risk of unprotected sexual behaviors and impairs sexual health decision-making. Furthermore, drinking alcohol prior to sexual intercourse was believed to provide a socially acceptable excuse for non-use of condoms, thereby, increasing the chance of getting into risky sexual behavior^28^.

Students who ever smoked cigarette were nearly two times more likely to be engaged in risky sexual behavior as compared to their counterparts. A similar finding has been reported from a previous study carried out in Adama town, Ethiopia which shows that those who smoked cigarettes were twce more likely to engage in risky sexual behavior than those who didn't smoke^32^. A possible explanation for the observed association may be due to the fact that students who smoke cigarettes may engage in risky sexual behavior due to observing such behaviors among their family or peers, or in the media. For instance, as has been reported elsewhere^33^, a substantial proportion of smokers in movies also engage in other risky behaviors, perhaps as part of portraying an image of “toughness” or “rebelliousness”.

Our study further identified the need for parental monitor in which the odds of reporting RSB among participants who had no parental monitor were around 90% higher than those who had parental monitor. There is a similar research conducted in Gondar^17^ and Haramaya town^26^ Ethiopia that shows youth who had good communication with their parents were less likely to engage in risky sexual activity.. This could be explained by the assumption that lack of opportunity for parental monitoring and guidance will provide the opportunity of being free from parental supervision so that, the students will have freedom of exercising sexual issues. As has been indicated elsewhere in this document, having close friend/s that are sexually active increases the chance for engaging in risky sexual behavior and parental monitoring may limit adolescents' exposure to high-risk peers. It has been well documented in the adolescent health literature that, Adolescents select friends who share similar attitudes, values, and behaviors and perceived peer norms and behavior are also strongly associated with risky sexual behavior. Sexual behavior is one of the many areas in which teens are influenced by their best friends and peers^24^,^27^. As well, this current study illustrates that; having close friend/s that are sexually active increases 59% the chance for engaging in risky sexual behavior as compared to their counterparts. This finding was comparable with the findings of other studies conducted among high school students in Gondar city, Ethiopia^17^ and youths at central Tanzania^31^. One possible explanation for the observed association could be that increased trust with their friends, level of peer influence on sexual matter, and by sharing information (including romantic and pornographic films) that may enhance the curiosity of sexual issues.

### Study limitation

The study findings should be viewed in light of the study limitations. First, since sexual behaviors remain very sensitive topics in Ethiopia, the resulting shame about reporting this information could have led to an underestimation of risky sexual behavior in the study area. Second, the questionnaires were distributed only to students present in the school. Since cigarette use, alcohol use, and sexuality may correlate with irregular attendance at school, lack of data from absent students may result in a lower representation of these groups. Third, because of the cross-sectional nature of our data, we are not able to draw conclusions about the causal effect and therefore, any generalization from the results of this study should be made with caution. Despite these limitations, our study demonstrates many strengths including a large number of students who were willing to participate in the study.

## Conclusion

This study has shown that almost one in five of study participants engaged in risky sexual behavior and that ever drink alcohol, ever smoked cigarette, parental monitoring and having sexually active close friend/s were factors significantly associated with risky sexual behavior among participants in the study area calling for more interventions on school students addictive behaviors.

Therefore, attempts need be made to limit youth access to alcohol and cigarettes through anti-tobacco marketing and smoking cessation campaigns mentioning the adverse health effects and encouraging parental and school community efforts to prevent alcohol drinking among students. Parents should have frequent, open and informative discussions about substance use and the associated problems with their adolescents. Moreover, parent monitoring and support towards academic and recreational activities is warranted. Furthr, it is important to educate students at school and in the community level to develop competencies that allow them to resist peer pressure.

## Abbreviations

RSB: Risky Sexual Behavior; SSA: sub-Saharan Africa; STDs: Sexually Transmitted Diseases; STIs: Sexually Transmitted Infections; WHO: World Health Organization;

## Declarations

### Availability of data and material

All data are already described and included in the manuscript
